# Health-related quality of life at 2, 6 and 12 months after critical illness - lessons learnt from a nationwide follow-up of 4,500 ICU admissions

**DOI:** 10.1186/2197-425X-3-S1-A408

**Published:** 2015-10-01

**Authors:** L Orwelius, E Åkerman, C-J Wickerts, SM Walther

**Affiliations:** Department of Clinical and Experimental Medicine, Linköping University, Linköping, Sweden; Department of Intensive Care, University Hospital, Linköping, Sweden; The Swedish Intensive Care Registry, Karlstad, Sweden; Clinic of Intensive Care and Perioperative Medicine, Skåne University Hospital (Malmö), Malmö, Sweden; Department of Medical and Health Sciences, Linköping University, Linköping, Sweden; Department of Cardiothoracic Anaesthesia and Intensive Care, University Hospital, Linköping, Sweden

## Introduction

The development of intensive care medicine has led to improved survival of patients with complex illnesses and extensive injuries. Survivors are at risk of acquiring physical and functional deficits that may have negative effects on health-related quality of life (HRQoL). The significance of measuring HRQoL has been underlined by critical care researchers since poor HRQoL is associated with an adverse prognosis.

## Objective

The aim of this work was to examine the development of HRQoL at 2, 6 and 12 months after ICU discharge in a mixed ICU patient population with an ICU-stay > 96 hrs.

## Methods

We analysed admissions during 2008-2014 to 49 ICUs that submitted follow-up data to the Swedish Intensive Care Registry (SIR, http://www.icuregswe.org). HRQoL was measured using the Short Form 36 (SF36) questionnaire at 2, 6, and 12 months after discharge from ICU. SF36 domains, age, gender, illness severity on admission (SAPS3 probabilities) and length of ICU-stay were analysed for the entire cohort and for important diagnostic groups. SF36 scores were compared to an age- and gender-adjusted Swedish normal population. Differences in SF36 domains were analysed using non-parametric methods. Medians and interquartile ranges are presented.

## Results

Complete SF36 responses were analysed for 4453, 4019 and 2515 admissions at 2, 6 and 12 months, respectively. HRQoL at 2 months in patients that subsequently were lost to follow-up was generally similar to those with follow-up, but they were younger, less ill and had shorter ICU-stay. Full longitudinal data with complete SF36 responses were obtained in 1438 patients [Age: 66 yrs. (57-73 yrs.), female gender: 37.2%, SAPS3 prob: 0.36 (0.19-0.55), ICU-stay: 7.0 days (4.9-11.5 days)]. SF36 improved over time in all domains (P < 0.001, Table), although some domains remained stable from 6 to 12 months. Patterns of recovery differed between important diagnostic groups (i.e. sepsis, out-of-hospital cardiac arrest, COPD, ARDS). A large proportion of patients (10-25% depending on SF36 domain) had HRQoL scores at 12 months which was below 2 standard deviations of the age- and gender-adjusted Swedish norm. The cardiac arrest group were among those with best, and the COPD group were among those with worst HRQoL at 12 months.

## Conclusions

HRQoL recovered over 12 months in critically ill patients with a prolonged ICU stay. Recovery varied between diagnostic groups and a large proportion of patients had markedly depressed HRQoL. These findings may have important implications for follow-up and care after critical illness.

## Grant Acknowledgment

The Swedish Intensive Care Registry is partly funded by grants from the Executive Committee of Swedish Quality Registries.

**Table 1:** Longitudinal HRQoL (SF36) after discharge from ICU.

	Physical function	Role Physical	Bodily Pain	General Health	Vitality	Social Function	Role Emotional	Mental health
2 mths (N = 1438)	50 (25-75)	0 (0-25)	52 (32-84)	54 (37-72)	45 (25-60)	63 (38-88)	33 (0-100)	72 (52-88)
6 mths (N = 1438)	65 (40-85)	25 (0-100)	62 (41-100)	57 (40-77)	55 (35-70)	75 (50-100)	100 (0-100)	80 (60-92)
12 mths (N = 1438)	70 (40-85)	25 (0-100)	62 (41-100)	57 (35-77)	55 (35-75)	75 (50-100)	100 (0-100)	80 (60-92

